# Association between underexpression of microrna-203 and clinicopathological significance in hepatocellular carcinoma tissues

**DOI:** 10.1186/s12935-015-0214-0

**Published:** 2015-06-18

**Authors:** Yongru Liu, Fanghui Ren, Minhua Rong, Yihuan Luo, Yiwu Dang, Gang Chen

**Affiliations:** Department of Pathology, First Affiliated Hospital of Guangxi Medical University, 6 Shuangyong Road, Nanning, Guangxi Zhuang Autonomous Region 530021 P. R. China; Research Department, Affiliated Cancer Hospital, Guangxi Medical University, 71 Hedi Road, Nanning, Guangxi Zhuang Autonomous Region 530021 P. R. China

**Keywords:** MiR-203, HCC, Adjacent non-cancerous liver, RT-qPCR, Metastasis

## Abstract

**Background:**

Although recent studies have shown the utility of miR-203 as a cancer-relevant biomarker, the validated clinical significance of miR-203 in HCC remains obscure. The aim of the present study was to evaluate the relationship between miR-203 expression and clinicopathological features in HCC patients.

**Methods:**

MiR-203 expression in 95 formalin-fixed, paraffin embedded (FFPE) HCC tissues and their paired adjacent non-cancerous tissues was evaluated by quantitative reverse transcription polymerase chain reaction (qRT-PCR). Simultaneously, expression of miR-203 and its correlation with a variety of clinicopathological parameters and patient recurrence was analyzed.

**Results:**

The relative level of miR-203 was 1.1651 ± 0.70378 in HCC tissues, significantly lower than its expression in the corresponding adjacent non-cancerous liver tissues (2.2408 ± 0.75351, *P* < 0.001). The area under curve (AUC) of low miR-203 expression to diagnose HCC was 0.85 (95 % CI: 0.796 ~ 0.904, *P* = 0.027) at a cut-off value 1.99 evaluated by the median expression of miR-203 in all tissues, including HCC and normal liver tissues. Expression of miR-203 was negatively correlated to metastasis (r = −0.254, *P* = 0.013), clinical tumor nodes metastasis (TNM) stage (r = −0.300, *P* = 0.003), nm23 expression (r = −0.292, *P* = 0.004), p21 expression (r = −0.223, *P* = 0.030), microvessel density (MVD)(r = −0.206, *P* = 0.045) and was positively correlated to cirrhosis (r = 0.487, *P* < 0.001). Additionally, the recurrent time of lower miR-203 expression group was 57.949 ± 4.184 months, slightly longer than that in the high expression group (54.682 ± 2.591 months), however, no significant difference was noted (Chi-square = 0.206, *P* = 0.650).

**Conclusions:**

MiR-203 plays a vital role in the carcinogenesis and progression of HCC, which makes itself as a predictor for the deterioration of HCC. Furthermore, miR-203 may become a new target for molecular therapy in HCC.

## Background

Hepatocellular carcinoma (HCC), with a 5-year survival rate of only 9 %, is the fifth most frequent malignancy and the third most common cause of cancer mortality worldwide [1–3]. The incidence of HCC is higher in male compared to the female. Among all cases, over 80 % of HCC patients occur in East Asia and sub-Saharan Africa, meanwhile, the incidence has increased substantially in Europe and the United States. Studies have shown that HCC resulted in 650,000 or more deaths per year all over the world, among which, three quarters occurred in East Asian countries [4, 5]. Multiple etiological factors contribute to the carcinogenesis and progression of HCC, for instance, infection of hepatitis B virus (HBV) or hepatitis C virus (HCV) and long-term exposure to some chemical agents (alcohol or aflatoxin B1) [6–9]. During the process of HCC development, gene expression, cell apoptosis, cell migration, vaso-invasion and capsule infiltration play essential roles [10]. HCC patients with advanced TNM stages or metastasis face a high incidence of recurrence and a dismal outcome [11]. It arouses our interest to improve the prognosis, to enhance the survival and to reduce the recurrence rate of HCC. Therefore, it is of vital importance to identify reliable biomarkers for the prediction of HCC and its recurrence along with to elucidate its role in HCC treatment.

MicroRNAs (miRNAs), an abundant class of endogenous, small, non-coding RNAs, have been identified as gene expression regulators [12, 13]. By binding to partially complementary or fully complementary sites within the 3′-untranslated region (UTR) of target messenger RNAs (mRNAs), miRNAs can trigger the translational repression, or deadenylation and degradation of mRNA [14, 15]. Recent studies have implicated that miRNAs are involved in a wide range of important biologic processes and essential tumorigenesis, including cellular growth and differentiation, developmental timing, modulation of host response to viral infection, proliferation, invasion and apoptosis [9, 16, 17].

Previous studies have indicated that microRNA-203 (miR-203) exhibited significantly down-regulated expression in prostate cancer [18], hematopoietic malignancy [19], colon cancer [20]. Other studies have shown that the low level expression of miR-203 played an important role in cell proliferation in the human head and neck squamous cell carcinoma, chronic myelogenous leukemia and B cell leukemia [21]. Concerning HCC, one study based on liver cancer cell lines identified that miR-203 was a tumor-suppressor gene silenced through tumor-specific DNA methylation in HCC [22]. Another study found that expression of miR-203 was low in tumor tissues of patients with post-LT (liver transplantation) HCC with recurrence compared to those in patients with non-recurrence [23]. However, aberrant expression of miR-203 between HCC and the paired non-cancerous liver tissues, as well as its clinicopathological significance in HCC has not been documented. Therefore, we aimed to quantify miR-203 expression in HCC tissues and compare it with the matched adjacent normal tissues in order to investigate the correlation between miR-203 expression and clinicopathological features, especially metastasis and recurrence in HCC patients.

## Results

### Characteristics of the HCC patients

The clinicopathological features of 95 HCC patients were shown in Table 1. All patients only underwent liver resection and nobody received any local ablative therapy (percutaneous ethanol injection PEI, radiofrequency ablation RFA), transhepatic arterial chemotherapy and embolization (TACE) or transhepatic arterial radioembolization (TARE) before liver resection. The majority of patients had well-differentiated or moderate-differentiated HCC while 29 patients (30.53 %) were poor-differentiated. Tumor sizes ranged from 1 to 11 cm (mean size, 6.4 cm). Vascular invasion was presented in 36 patients (37.89 %) while 50 patients presented with tumor capsule infiltration (52.63 %). Thirty-eight patients (40 %) had elevated serum alpha fetal protein (AFP) levels (400 ng/ml). The mean time of follow-up was 33.13 ± 1.71 months (range: 2.68–68.00 months). Of the 70HCC patients included in the follow-up, 59 had recurrent tumors and 11 were dead or censored at the end of follow-up.

**Table 1:** Relationship between miR-203 expression and clinicopathological features in HCC

Clinicopathological Features		*N*	miRNA-203 relevant expression (2^-ΔCq^)		
			Mean ± SD	*t*	*P*
Tissue	Adjacent non-cancerous liver	95	2.2408 ± 0.75351	10.170	<0.001
	HCC	95	1.1651 ± 0.70378		
Age	<50	49	1.0951 ± 0.72640	1.000	0.320
	≥50	46	1.2396 ± 0.67884		
Gender	male	75	1.1163 ± 0.70032	−1.313	0.192
	female	20	1.3480 ± 0.70405		
Differentiation	well	6	1.1717 ± 0.53229	0.804^*a*^	0.451
	moderate	60	1.2305 ± 0.73598		
	poor	29	1.0283 ± 0.66507		
Size	<5 cm	18	0.9061 ± 0.50149	−2.208	0.034
	≥5 cm	77	1.2256 ± 0.73265		
Tumor nodes	single	52	1.2960 ± 0.76903	2.026	0.046
	multiple	43	1.0067 ± 0.58613		
Metastasis	-	46	1.3363 ± 0.77316	2.353	0.021
	+	49	1.0043 ± 0.59589		
Clinical TNM stage	I-II	22	1.5600 ± 0.79292	3.141	0.002
	III-IV	73	1.0460 ± 0.63345		
Portal vein tumor embolus	-	63	1.2387 ± 0.76142	1.440	0.153
	+	32	1.0200 ± 0.55646		
Vaso-invasion	-	59	1.1810 ± 0.76883	0.282	0.779
	+	36	1.1389 ± 0.59126		
Tumor capsular infiltration	With complete capsule	45	1.1549 ± 0.75314	−0.133	0.895
	Infiltration or no capsule	50	1.1742 ± 0.66383		
HCV	-	63	1.1073 ± 0.71115	−1.124	0.264
	+	32	1.2788 ± 0.68580		
HBV	-	17	1.2624 ± 0.51125	0.627	0.532
	+	78	1.1438 ± 0.74014		
AFP	-	41	1.1173 ± 0.69725	−0.629	0.531
	+	38	1.2184 ± 0.73104		
Cirrhosis	-	50	1.4912 ± 0.72123	5.436	0.000
	+	45	0.8027 ± 0.47296		
MTDH	-	38	1.1561 ± 0.69533	0.162	0.872
	+	51	1.1320 ± 0.69657		
nm23	-	20	1.5965 ± 0.82139	3.237	0.002
	+	75	1.0500 ± 0.62628		
p53	-	40	1.1412 ± 0.69109	−0.280	0.780
	+	55	1.1824 ± .71871		
p21	-	62	1.2758 ± 0.72648	2.142	0.035
	+	33	0.9570 ± 0.61683		
VEGF	-	25	1.4252 ± 0.82009	2.196	0.031
	+	70	1.0721 ± 0.63841		
Ki-67 LI	Low	47	1.1726 ± 0.76271	0.102	0.919
	High	48	1.1577 ± 0.64897		
MVD	Low	47	1.1900 ± 0.69824	0.340	0.734
	High	48	1.1406 ± 0.71570		

*N* number, *SD* standard deviation, *TNM* tumor node metastasis, *HBV* hepatitis B virus, *HCV* hepatitis C virus, *AFP* alpha fetal protein, *MTDH* metadherin, *VEGF* vascular endothelial growth factor, *LI* labeling index, *MVD* microvessel density

^a^ANOVA test was performed

### Value of miR-203 in the diagnosis of HCC

**Fig. 1:**
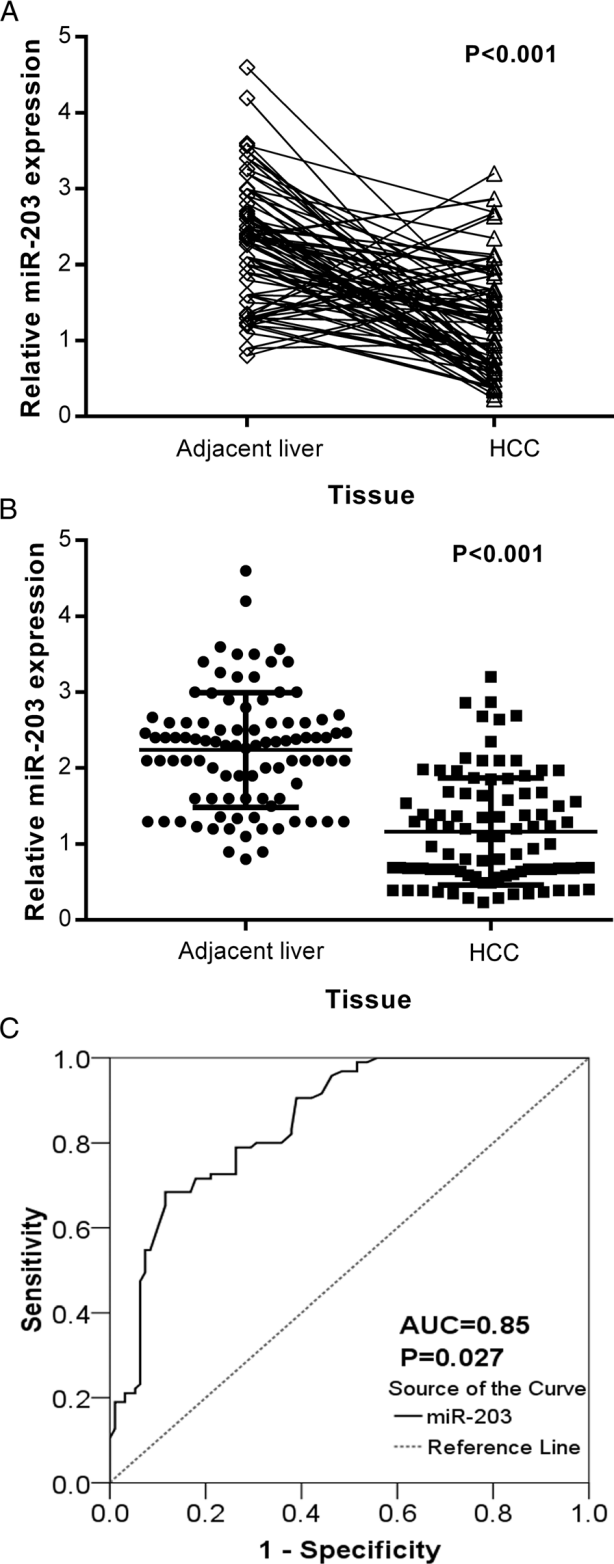
Expression of miR-203 in adjacent non-cancerous liver and HCC tissues. Quantitative real-time PCR was performed to detect the expression of miR-203. **a**: Relative expression of miR-203 in adjacent non-cancerous liver and HCC tissues (dot plots with lines linking each pairs). **b**: The difference of relevant miR-203 expression between adjacent non-cancerous liver and HCC tissues. *** *P* **<** 0.001. **c**: ROC curve of miR-203 expression to distinguish HCC from non-cancerous liver. The area under curve (AUC) of miR-203 was 0.85 (95 % CI: 0.796 ~ 0.904, *P* = 0.027) at a cut-off value of 1.99. Error bars represented standard deviation (SD). The statistical analysis was performed with Student’s *t*-test (**a** and **b**)

There was a significant difference of relative miR-203 expression between HCC and the paired adjacent non-cancerous liver tissues. The relative level of miR-203 was 1.17 ± 0.70 in HCCs, significantly lower than that of the adjacent non-cancerous liver tissues (2.24 ± 0.75, *P* < 0.001, Fig. 1a, b). Additionally, we conducted ROC curve to identify the diagnostic role of miR-203 in HCC. The area under curve (AUC) of miR-203 was 0.85 (95 % CI: 0.796 ~ 0.904, *P* = 0.027, Fig. 1c) at a cut-off value of 1.99.

### The relationship between miR-203 expression and clinicopathological parameters of HCC

**Fig. 2:**
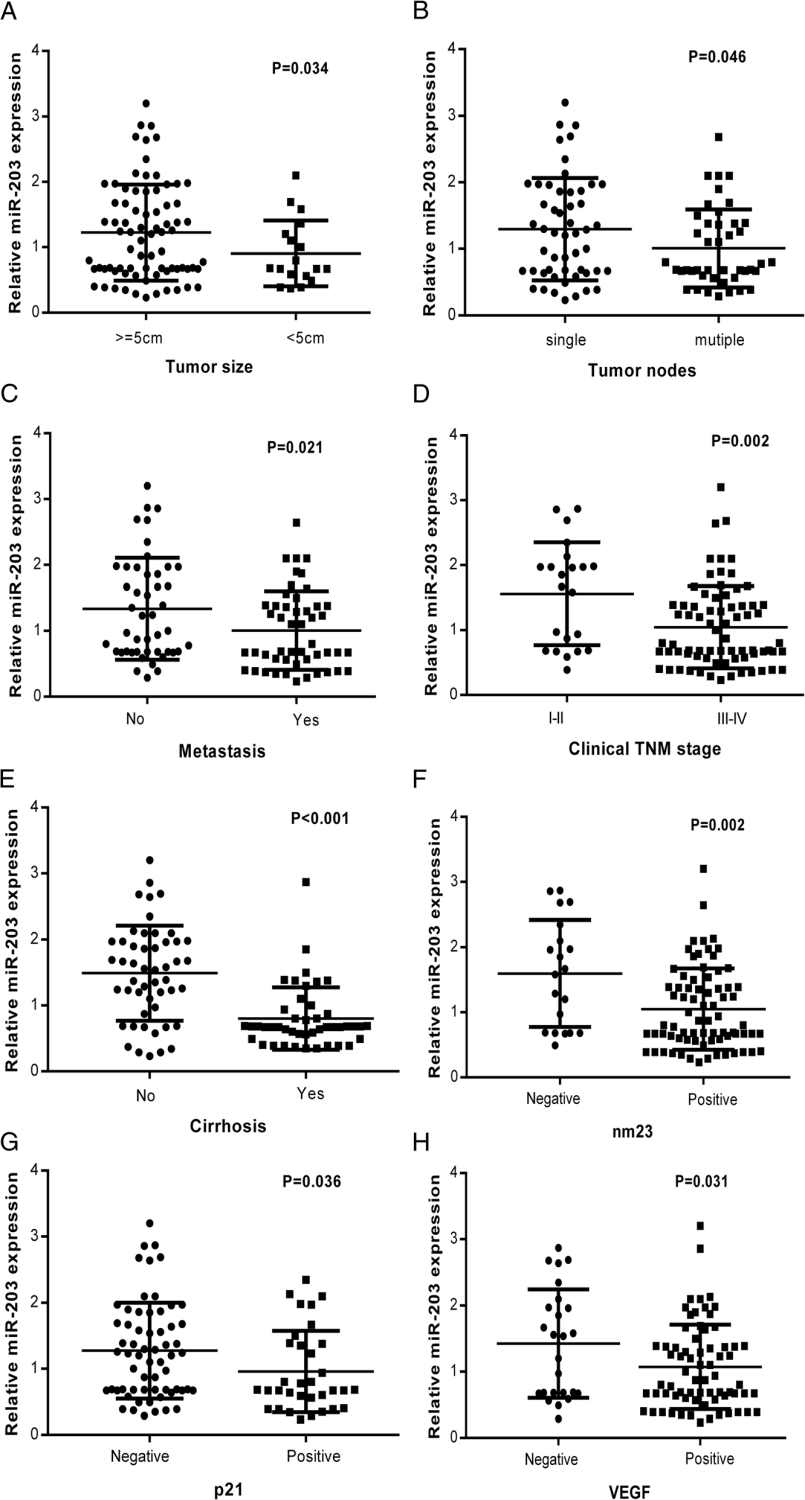
The relationship between miR-203 expression and clinicopathological parameters of HCC. **a**: Tumor size, (**b**): Tumor nodes, (**c**): metastasis status, (**d**): clinical TNM stage, (**e**): cirrhosis, (**f**): nm23, (**g**): p21, (**h**): VEGF. The data were representative of two independent experiments. Error bars represented SD. * *P* **<**0.05, ** *P* **<**0.01, *** *P* **<**0.001 by Student’s *t*-test (**a-g**)

**Fig. 3:**
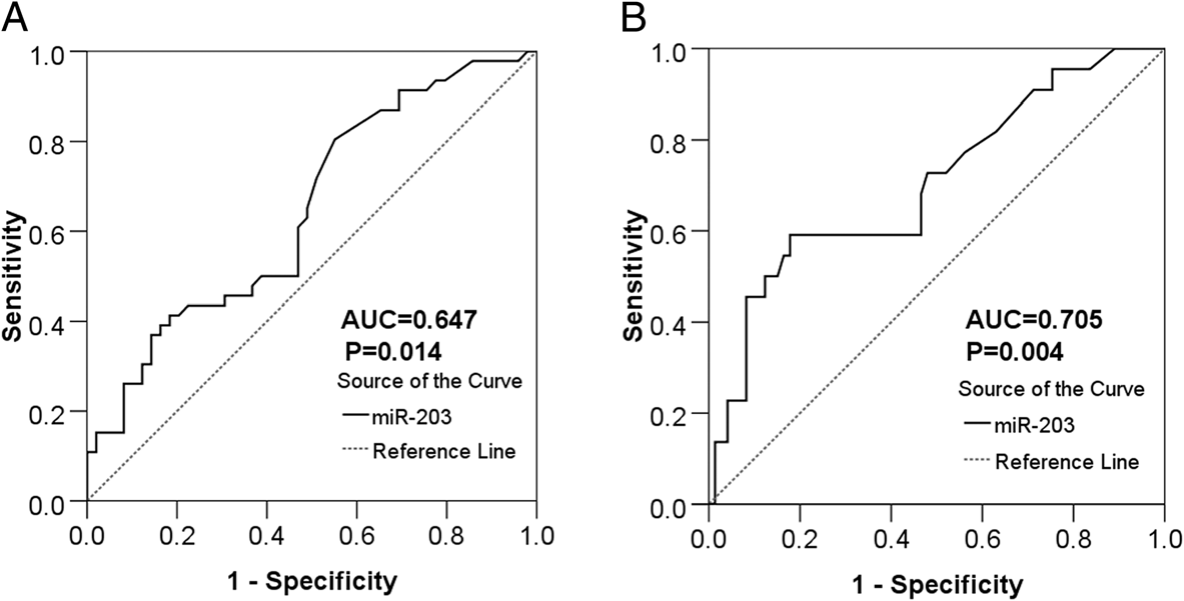
Diagnostic significance of different parameters on HCC. **a**. The ROC curve of miR-203 for discriminating metastasis from non-metastasis in HCC patients. The AUC of miR-203 and metastasis was 0.647 (95 % CI: 0.536 ~ 0.757, *P* = 0.014) at a cut off value of 1.52. **b**. ROC curve of miR-203 expression to distinguish advanced clinical TNM stage from early TNM stage in patients with HCC. The AUC of miR-203 and TNM was 0.705 (95 % CI: 0.575 ~ 0.834, *P* = 0.004) at a cut-off value of 1.57. The cut off values were measured according to the median expression of miR-203 in HCC tissues. Error bars represented SD. Significance of difference between two groups was analyzed by Student’s *t* test (**a** and **b**)

With respect to the association between miR-203 expression and clinicopathological parameters of HCC, the following results were obtained. The relative expression of miR-203 in HCC patients with tumor larger than 5 cm (1.23 ± 0.73) was remarkably higher in comparison with those whose tumor were smaller than 5 cm (0.9061 ± 0.50149, *P* = 0.034). Also, the relative expression of miR-203 in HCC patients with single tumor nodes (1.30 ± 0.77) was prominently higher than those with multiple tumor nodes (1.01 ± 0.59, *P* = 0.046). Compared to those with metastasis (1.00 ± 0.60), the level of miR-203 was higher in those HCC patients without metastasis (1.34 ± 0.77, *P* = 0.021). When compared with HCC patients of advanced stages (III and IV, 1.05 ± 0.63), the relative expression of miR-203 in early stages patients (I and II, 1.56 ± 0.79, *P* = 0.002) was notably increased. Besides, the relative expression of miR-203 in HCC patients without cirrhosis (1.49 ± 0.72) was obviously higher than those with cirrhosis (0.80 ± 0.47, *P* < 0.001). In association with nm23, HCC patients with negative expression of nm23 (1.60 ± 0.82) had a higher level of miR-203 than those with positive expression (1.05 ± 0.63, *P* = 0.002). As for the relationship with the expression of p21, negative expresser had a noticeably higher level of miR-203 (1.28 ± 0.73) than the positive one (0.96 ± 0.62, *P* =0.035). Moreover, miR-203 level in VEGF negative HCC patients was higher (1.43 ± 0.82), compared with VEGF positive HCC patients (1.07 ± 0.64, *P* = 0.031, Fig. 2, Table 1). Meanwhile, we conducted a further analysis using Spearman correlation test, the consistent relationship between the expression of miR-203 and the clinicopathological parameters of HCC were listed as follows: metastasis (*r* = −0.254, *P* = 0.013), TNM (*r* = −0.300, *P* = 0.003), cirrhosis (*r* = 0.487, *P* < 0.001), nm23 (*r* = −0.292, *P* = 0.004), p21 (*r* = −0.223, *P* = 0.030) and MVD (*r* = −0.206, *P* = 0.045). Additionally, the AUC of miR-203 and metastasis was 0.647 (95 % CI: 0.536 ~ 0.757, *P* = 0.014, Fig. 3a), and the AUC of miR-203 and TNM was 0.705 (95 % CI: 0.575 ~ 0.834, *P* = 0.004, Fig. 3b). However, no association was found between miR-203 expression and other clinicopathological features, such as age, gender, tumor differentiation grades, portal vein tumor embolus, vascular invasion, tumor capsular infiltration, HCV or HBV infection condition, AFP, metadherin (MTDH), p53, vascular endothelial growth factor (VEGF) and ki-67 expression. As for tumor size and tumor nodes, expression of miR-203 between each two groups was significantly different, respectively. However, no correlation was found between miR-203 and tumor size or tumor nodes by Spearman analysis.

### Role of miR-203 expression in recurrence of HCC

Among all the 95 patients, we had a successful follow-up of 70 patients, among which 52 had low miR-203 expression (lower than the median level of 0.97) while 18 had high miR-203 expression. As for time to recurrence, low miR-203 expression group was 57.949 ± 4.184 months, while the high expression group was 54.682 ± 2.591 months. No significant difference of recurrent time was found between the low and high miR-203 groups (Chi-square = 0.206, *P* = 0.650).

## Discussion

Up till now, there have been only three studies that explored the role of miR-203 in HCC [22–24]. Among the available articles, Wang et al. [24] only evaluated expression of miR-203 in HepG2 cells by Western blot assay without using clinical HCC specimens. Furuta et al. [22] examined a total of 19 liver cancer cell lines and 41 frozen primary tumor samples with their corresponding non-tumorous tissues and confirmed that expression of miR-203 was silenced through tumor-specific DNA methylation in HCC. Chen et al. [23] confirmed that expression of miR-203 was lower in tumor tissues. Chen et al. reported that miR-203 expression was measured in 66 HCC patients who underwent orthotopic deceased donor liver transplantation. However, the 95 HCC patients who were selected into the current study received no additional treatment including chemoradiotherapy, liver transplantation or ablation. Of note, our current study collected and compared 95 HCC samples with their paired adjacent non-cancerous tissues twice more than the 41 samples examined in the research of Furuta et al. [22]. Of particular note, these paired samples eliminated the individual differences and achieved better comparability. As a result, significantly lower level of miR-203 expression was found in HCC tissues compared with adjacent non-cancerous tissues. The ratio of miR-203 in HCC tissues to that in non-cancerous tissues was 0.52. Furthermore, the AUC of 0.85, nearly 0.9 from ROC analysis demonstrated that miR-203 had a specific diagnostic value for HCC. Combined with the literatures, our current finding strongly indicated that miR-203 could be regarded as a tumor-suppressor in HCC.

**Fig. 4:**
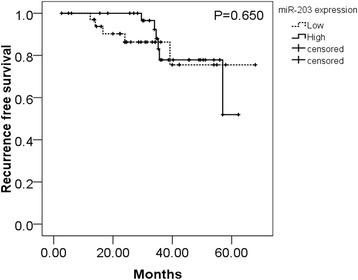
Recurrence free survival of HCC patients according to miR-203 levels. There was no significant association between miR-203 expression and recurrence free survival in patients with HCC (*P* = 0.650). Error bars represented SD. Survival difference according to miR-203 expression was analyzed by the log-rank test

We primarily revealed that expression of miR-203 was relevant to the deterioration of HCC by analyzing the association between miR-203 expression and clinicopathological parameters. First of all, miR-203 was down-regulated in HCC tissues with multiple tumor nodes and with metastasis, as compared to their corresponding traits respectively. Furthermore, miR-203 expression was negatively related to HCC progression. MiR-203 reduced evidently when there appeared cirrhosis or when HCC developed into advanced stage. In addition, expression of miR-203 was down-regulated in HCC tissues where nm23 and p21 were positive. Above results indicated that low level of miR-203 was closely associated with factors of HCC development, such as metastasis, multiple tumor nodes and late clinical stages. However, miR-203 was of higher level in HCC tissues whose tumor sizes were over 5 cm. Under normal conditions, we regard larger size tumors as the more aggressive ones. Here, the result was contradictory. MiR-203 may play more important roles in tumor cell invasion and metastasis than tumor cell growth. However, this hypothesis needs further *in vitro* and *in vivo* experiments to confirm in the future. For the relationship between miR-203 level and patient recurrence, although patients with lower level of miR-203 showed a longer time of recurrence than the higher level patients, no statistical significance was noted (Fig. 4). Cohorts with larger patient size are required to figure out the correlation between miR-203 and recurrence in HCC.

MVD, VEGF and MTDH are factors related to angiogenesis, which play a vital role in tumor growth and progression. Among them, MTDH was found to have partly complementary sequences with miR-203 with several prediction software (miRbase, Tarbase, targetScan and miRanda). Li et al. [25] have demonstrated that by negatively regulating Akt2 protein expression, miR-203 reversed chemoresistance in p53-mutated colon cancer cells where MTDH was involved. Zhu et al. [26] pointed out that miR-203 down-regulated vascular endothelial growth factor alpha (VEGFA) expression by directly targeting its 3′-untranslated region in human cervical cancer. Nevertheless, we found no significant correlation between miR-203 and MTDH or VEGF in HCC, only MVD showed a significant correlation with miR-203 (*r* = −0.20607 *P* = 0.045). Other cohort with more patients involved and *in vitro* study are required to examine the potential role of MTDH as a target of miR-203. As Furuta et al. [22] suggested in his article, the silencing of miR-203 contributed to the pathogenesis of HCC by activating of ATP binding cassette E1 (ABCE1).

And Wang et al. [24] revealed that low expression of miR-203 dedicated to the progression of HCC via targeting survivin. All these findings strongly support our result that expression of miR-203 was relevant to the deterioration of HCC via different pathways.

## Conclusion

In accordance with previous researches, our current study strongly prompts that miR-203 is a tumor-suppressive miRNA, contributing to the carcinogenesis and deterioration of HCC. Expression of miR-203 can be a diagnostic biomarker for HCC. To investigate and identify the function of miR-203 in HCC, as well as to regard miR-203 as a new target for HCC treatment, further *in vitro* and *in vivo* studies are needed in future.

## Methods

### Patients and tissue samples

A total number of 95 HCC patients (75 males and 20 females, with a mean age of 52 years old, ranged from 29 to 82 years old), who were admitted to the First Affiliated Hospital of the Guangxi Medical University (Nanning Guangxi, China) between March, 2010 and December, 2011, were selected for this current study. Their formalin-fixed, paraffin embedded (FFPE) tumor tissues and adjacent non-cancerous liver tissues were retrospectively evaluated. The clinicopathological parameters of patients including age, gender, differentiation, tumor size, tumor nodes, metastasis and clinical TNM stages, the presence or absence of portal vein tumor embolus, vaso-invasion, capsular infiltration and cirrhosis, and other biomarkers such as serum AFP level, expression of metadherin (MTDH), nm23, p53, p21, vascular endothelial growth factor (VEGF) and microvessel density (MVD). The clinical TNM stage is the most common system for staging to employ the TNM classification. A “T” score is based upon the size and/or extent of invasion. The “N” score indicates the extent of lymph node involvement. The “M” score indicates whether distant metastasis is present. All these features were summarized in Table 1. The Ethical Committee of First Affiliated Hospital, Guangxi Medical University, China approved the current research, and informed consent was obtained from all participating patients. Related research was conducted in accordance with the Helsinki Declaration. All samples were reviewed and diagnosed by two independent pathologists.

### RNA isolation and quantification of miR-203

Extraction and normalization of RNAs along with miRNAs by using the miRNeasy FEPE Kit (QIAGEN, KJ Venlo, Netherlands) were performed as described previously [27–30]. RNA concentrations were determined by NanoDrop 2000 (Wilmington, DE, USA). A combination of RUN6B and RUN48 was the housekeeping gene for detection of miR-203 expression in HCC FFPE tissues [27, 28]. The primers for miR-203, RNU6B and RNU48, were included in TaqMan®MicroRNAAssays (4427975, Applied Biosystems, Life Technologies, Grand Island, NY, USA). Sequence of miRNA and references used in the paper are as follows: miR-203 (Applied Biosystems Cat. No. 4427975–000507): GUGAAAUGUUUAGGACCACUAG; RNU6B (Applied Biosystems Cat. No. 4427975–001093): CGCAAGGAUGACACGCAAAUUCGUGAAGCGUUCCAUAUUUUU; RNU48 (Applied Biosystems Cat. No. 4427975–001006): GAUGACCCCAGGUAACUCUGAGUGUGUCGCUGAUGCCAUCACCGCAGCGCUCUGACC. The reverse primers were also used for reverse transcription with TaqMan®MicroRNA Reverse Transcription Kit (Amsterdam, Netherland, Applied Biosystems, Life Technologies) in a total volume of 10 μL. Real-time qPCR for miRNA was performed with Applied Biosystems PCR7900. The miR-203 abundance in each sample was normalized to its references. The expression of miR-203 in the FFPE experiments was calculated with the formula 2^-Δcq^ [27–31].

### Statistical analysis

SPSS 20.0 (Munich, Germany) was used for statistical analysis. Quantitative variables were presented as means ± standard deviation (SD). Significance of difference between two groups was analyzed by Student’s *t* test. Significance of difference within three groups (we divided differentiation condition into three groups) was analyzed by One-way analysis of variance (ANOVA) test. Correlation between miR-203 expression and clinicopathological parameters was detected by using Spearman. Effectiveness of miR-203 to distinguish HCC from non-cancerous liver tissues was generated by receiver operating characteristic (ROC) curves. Recurrence analysis was estimated by the Kaplan–Meier method, and the log-rank test was used to compare the recurrence between groups. When *P*-value was less than 0.05, calculated by two-tailed test, it was considered as statistically significant.

## Abbreviations

HCC: Hepatocellular carcinoma
HBV: Hepatitis B virus
HCV: Hepatitis C virus
TNM: Tumor node metastasis
Clinical TNM stage: A “T” score is based upon the size and/or extent of invasion. The “N” score indicates the extent of lymph node involvement. The “M” score indicates whether distant metastases are present
QRT-PCR: Quantitative reverse transcription polymerase chain reaction
AUC: Area under curve
miRNAs: MicroRNAs
miR-203: MicroRNA-203
LT: Liver transplantation
PEI: Percutaneous ethanol injection
RFA: Radiofrequency ablation
TACE: Transhepatic arterial chemotherapy and embolization
TARE: Transhepatic arterial radioembolization
FFPE: Formalin-fixed, paraffin embedded
AFP: Alpha fetal protein
MTDH: Metadherin
VEGF: Vascular endothelial growth factor
MVD: Microvessel density
ANOVA: One-way analysis of variance
ROC: Receiver operating characteristic
L: Labeling index
